# Latin American and Caribbean countries’ baseline clinical and policy guidelines for responding to intimate partner violence and sexual violence against women

**DOI:** 10.1186/s12889-015-1994-9

**Published:** 2015-07-15

**Authors:** Donna E. Stewart, Raquel Aviles, Alessandra Guedes, Ekaterina Riazantseva, Harriet MacMillan

**Affiliations:** University Health Network, Centre for Mental Health, University of Toronto, Toronto, Canada; University Health Network, Toronto, Canada; Pan American Health Organization/World Health Organization Regional Office for the Americas, Washington, DC USA; Offord Centre for Child Studies, McMaster University, Hamilton, Canada

## Abstract

**Background:**

Violence against women is a global public health problem with negative effects on physical, mental, and reproductive health. The World Health Organization (WHO) has identified intimate partner violence (IPV) and sexual violence (SV) as major targets for prevention and amelioration and recently developed clinical and policy guidelines to assist healthcare providers. This project was undertaken to determine the 2013 baseline national policies and clinical guidelines on IPV and SV within the Latin American and Caribbean (LAC) region to identify strengths and gaps requiring action.

**Methods:**

Each Pan American Health Organization/World Health Organization Regional Office for the Americas (PAHO/WHO) country focal point was contacted to request their current national policy and clinical guidelines (protocol) on IPV/SV. We augmented this by searching the internet and the United Nations Women website. Each country’s policy and clinical guideline (where available) was reviewed and entered into a scoring matrix based on WHO Clinical and Policy Guidelines. A total score for each heading and subheading was developed by adding positive responses to identify LAC regional strengths and gaps.

**Results:**

We obtained 15 national policies and 12 national clinical guidelines (protocols) from a total of 18 countries (“response” rate 66.7 %). National policies were comprehensive in terms of physical, emotional, and sexual violence and recommended good intersectoral collaboration. The greatest gap was in the training of health-care providers. National Guidelines for women-centered care for IPV/SV survivors were strong in the vital areas of privacy, confidentiality, danger assessment, safety planning, and supportive reactions to disclosure. The largest gaps noted were again in training healthcare professionals and strengthening monitoring and evaluation of services.

**Conclusions:**

Baseline measurement of policy and clinical guidelines for IPV/SV in LAC PAHO/WHO member countries at the time of issuing the 2013 WHO Clinical and Policy Guidelines reveals some important strengths, but also serious gaps that need to be addressed. The most pressing needs are for concerted training initiatives for healthcare providers and strengthening multisectoral monitoring and evaluation of services. A future evaluation of national policies, clinical guidelines, monitoring and evaluation will need to be conducted to measure the progress of the required scaling-up process.

## Background

Violence against women is a global public health problem and a violation of human rights, which has substantial negative effects on physical, mental, and reproductive health, as well as detrimental social and economic impacts [1]. While there are several types of violence, the World Health Organization (WHO) has identified intimate partner violence (IPV) and sexual violence (SV) as major targets for prevention and amelioration [2]. A common theme for both IPV and SV is the use of violence as an expression of control, power and domination. Several definitions of these terms exist, but common ones for IPV include physical, sexual or psychological harm by a current or former partner or spouse, which can occur among heterosexual or same-sex partners and does not require sexual intimacy [3]. Sexual violence is any act perpetrated against someone’s will, including a completed non-consensual sex act (rape), an attempted non-consensual sex act, abusive sexual contact (e.g. unwanted touching) or noncontact sexual abuse (e.g. exhibitionism, threats of sexual violence, sexual harassment) [4].

The prevalence of IPV and SV varies internationally, partly due to different definitions and approaches to measurement. However, a WHO multicountry study (24,000 women) on domestic violence against women in 10 countries, using comparable data, found the highest lifetime prevalence (61 %) of physical IPV in rural Peru [5]. Other studies also report high rates of IPV in other Latin American [6, 7] and Caribbean countries [8]. The Pan American Health Organization/World Health Organization Regional Office for the Americas (PAHO/WHO) provides technical cooperation and mobilizes partnerships to improve health and quality of life in the countries of the Americas, including in Latin America and the Caribbean (LAC) [9].

In response to requests from Member States and as part of its efforts to prevent and ameliorate IPV and SV, WHO developed clinical and policy guidelines to assist healthcare providers, who are likely to be the first professional contact for survivors. To develop these clinical and policy guidelines an expert panel was assembled for a Guideline Development Group which included academics, clinicians/ service providers, policy makers, and women’s health and rights advocates from across the globe, including low- and middle-income countries. A literature review was undertaken and scientific evidence for recommendations was synthesized using the Grading of Recommendations Assessment, Development and Evidence (GRADE) methodology [10]. Recommendations were agreed upon by the Guideline Development Group and were then reviewed by peer reviewers and a wide range of stakeholders before the final report was released in July 2013 [11].

A useful first step in implementing the new WHO Clinical and Policy Guidelines is to determine the existing (baseline) national policies and clinical guidelines for IPV and SV to identify strengths and gaps. This project, supported by PAHO/WHO, was undertaken to determine the 2013 baseline national policies and clinical guidelines on IPV and SV within the LAC region.

## Methods

Four sources were used to obtain the maximum number of national policies and clinical guidelines in each LAC country member of PAHO in which the primary language was Spanish, English or Portuguese. Each PAHO/WHO country focal point was contacted in July 2013 by the PAHO/WHO Regional Advisor on Family Violence (AG) to inform them about this project, to introduce the trilingual study coordinator (RA), and to ask them to send their current national policy and clinical guidelines (protocol) on IPV/SV. This was followed up by the study coordinator with at least three reminders by email and phone to non-responders. We informed each country at the time of contacting them that we would not publish their individual results, but these would be available to PAHO. PAHO/WHO focal points were our primary source, but we augmented it by an internet search for national policy and clinical guidelines on IPV/SV for each LAC country, contact by one of the investigators with knowledgeable colleagues where available for any LAC country and searching the United Nations (UN) Women website for any documented LAC national policy on IPV/SV.

The WHO Clinical and Policy Guidelines were used to develop a scoring matrix for each country. The headings with appropriate subheadings included women-centered care for IPV/SV survivors, additional issues for SV survivors, and training of healthcare providers. The scoring matrix was reviewed and approved by five experts on IPV/SV/CM (child maltreatment), including three involved in developing the WHO Guidelines.

Each country’s policy and clinical guideline (where available) was reviewed and entered into the scoring matrix by the study coordinator along with the appropriate page number. Two investigators reviewed and approved the scoring matrix entries. The scoring matrix for each country was returned to the appropriate PAHO/WHO focal point who was asked to review and approve it (or provide a correction) in collaboration with their Ministry of Health. Two reminder emails were sent to non-responders. To preserve confidentiality of individual PAHO/WHO countries, a total score for each heading and subheading was developed by adding positive responses from all responding countries to identify LAC regional strengths and gaps requiring action. We defined large gaps as aggregate scores of 3 or less (out of 12), moderate gaps as aggregate scores of 4 to 7 and strengths as aggregate scores of 8 to 12. An overall report was written and submitted to PAHO/WHO.

## Results

We obtained 15 national policies and 12 national clinical guidelines (protocols) from a total of 18 countries (“response” rate of 66.7 %, but not all countries had both a policy and guideline). Countries that responded included Argentina, Belize, Bolivia, Brazil, Chile, Colombia, Costa Rica, Ecuador, El Salvador, Guatemala, Guyana, Honduras, Mexico, Nicaragua, Panama, Peru, Uruguay, Venezuela. Seven countries were “non-responders” despite at least three reminders by email or phone and an internet search, and two countries sent information other than national policies or guidelines. Access to policies and guidelines was available to the public via an online version or the country’s Ministry of Health or Women’s Institute in all but 2 responding countries. The definitions of IPV, SV, CM by country varied slightly in wording, but all were consistent with those found in the WHO Guidelines. A few countries used the generic terms “domestic violence” or “family violence”, but the subsequent wording clarified the specific form of violence (IPV, SV or CM) being discussed.

### National policies

The time frame for the implementation of the 15 policies obtained ranged from 2006 to 2017 and all addressed physical, emotional and sexual violence against women. These were developed by the Ministry of Health (5), in collaboration with one or more other government sources (9), local or regional governments (1), professional associations (2), or other sources (7). Policies addressed sectors in health (15), education (13), justice or police (12), social development (4), media (3), work (3), human rights (1), public security (1), parliament (1) and sports (1). Most policies addressed violence prevention (14), how to respond to disclosure (15), in children and adolescents (14) and women survivors (15). Two countries recommended pre-qualification training and 12 in-service training for health care providers, however the frequency and length of training in all policies was unspecified. The content of training included when and how to respond to violence (3), best ways to respond (8), how to collect forensic evidence (2), specific information about violence against women (VAW) local laws (7), existing services (3), provider attitudes (5), and provider’s own experience of violence (1).

### National clinical guidelines

Of the 12 national guidelines (protocols) for the health sector that were obtained, 4 guidelines included IPV, SV and CM, 3 covered only SV, and 5 covered one or more of these topics, most commonly CM. Mandatory reporting was required for CM in 10, SV in 9 and IPV in 4 guidelines. If reporting was not mandatory, 10 guidelines recommended respecting the women’s decision about reporting. Ten guidelines differentiated between adult and child survivors of SV. Recommendations in the WHO Clinical Guidelines related to women-centered care for IPV/SV survivors were selectively addressed in many guidelines as shown in Table 1.

**Table 1:** Women-centered care for IPV/SV survivors (n = 12 guidelines)

Visual and auditory privacy during consultation	12
Confidentiality when information shared	12
Non-judgemental, supportive, validating reaction to disclosure	12
Safety planning or danger assessment	10
Assess and promote children’s safety	7
Provide written material about legal, housing, economic empowerment	8
Warn of risk to women of taking home written material	0
Special needs women: mental disorder	9
physical disability	8
pregnancy	6
Universal screening (NOT recommended by WHO)	4
Selective enquiry (recommended by WHO)	4
Referral for needed services	9
Referral pathway outlined	8
Availability of directory of existing services	3
Women’s shelter available	3
Hotlines available	3
Assess for mental health problems	7
suicide/self-harm	6
depression/anxiety	4
substance abuse	3
posttraumatic stress disorder	1
Provide or mobilize social support	6
Offer coping strategies for stress	0
Undertake watchful waiting to see if early distress resolves	7
Provide psychological first aid support	6
Offer structural advocacy/empowerment	6
Mental health service available	7
Offer psychotherapy for children exposed to VAW	4
Photos of injuries if present	5
Body diagram of injuries if present	7

Additional recommendations for SV survivors such as specific services, risk and management of pregnancy, sexually transmitted infections (STIs), human immunodeficiency virus (HIV), hepatitis B and forensic documentation are shown in Table 2.

**Table 2:** Additional issues for sexual violence survivors

Sexual assault services available 24/7	4
Record time since assault	8
Record type of assault	8
Vaginal swabs	8
Anal swabs	6
Oral swabs	6
Collect hair and fibers	5
Risk of pregnancy	8
Emergency contraception within 5 days SV	8
Levonorgestrel (1 or doses)	6
Combined estrogen/progesterone	5
Copper bearing IUD	1
Ulipristal acetate	0
Referral for abortion if legal and requested	4
Risk of STIs	8
STI testing before treatment (not recommended)	1
Chlamydia treatment	7
Gonorrhea treatment	7
Trichomonas treatment	7
Syphilis treatment	6
Hepatitis B vaccine offered (without immunoglobulin)	5
Risk of HIV	8
HIV PEP appropriateness counselling	6
Shared decision making for HIV PEP	5
Does not require HIV testing before PEP	12
Start HIV PEP within 72 h	6
HIV testing and counselling at initial consultation	8
Regular follow-ups	5
2 drug regime for HIV PEP	4
3 drug regime for HIV PEP	4
Prioritize drugs with fewer side effects	1
Drugs follow national guidelines	5
Adherence counselling offered	5

Training of healthcare providers on IPV/SV and the monitoring and evaluation of policy, guidelines and services are shown in Table 3.

**Table 3:** Training of healthcare providers

Prequalification training of doctors, nurses, midwives at university	0
In-service training for direct care providers	6
Where and how to enquire about violence	3
Best ways to respond to disclosure	5
How to collect forensic evidence	0
IPV/SV information	6
Local laws on IPV/SV	2
Existing services	3
Providers’ attitudes	3
Providers’experiences of violence	1
Length and frequency of training	1
Monitoring and evaluating services	2
Women survivors involved in evaluating services	0
Mechanism for intersectoral collaboration	5
Meetings promoted between sectors (legal, health, social)	4
Support for vicarious trauma to providers	2

## Discussion

The challenges we experienced in obtaining current national policies and clinical guidelines on IPV and SV in LAC are illustrative of some of the difficulties of conducting health and policy research in low- and middle-income countries. We acknowledge the limitation that one third of all countries approached did not respond. The low response rate limited researchers’ ability to carry out subregional comparisons. Nevertheless the information we obtained from the 66.7 % of countries we approached provides a useful baseline snap-shot of the strengths and gaps to inform the implementation of the July 2013 WHO Clinical and Policy Guidelines.

Policies:

National policies we obtained were fairly comprehensive in terms of covering physical, emotional, and sexual violence and some displayed good intersectoral collaboration by involving a number of government and civil sectors, which are vital for a strong government and community response to IPV/SV. The policies were commendably strong in addressing violence prevention and how to respond to IPV/SV in women and children in a supportive manner. The greatest gap was in the training of health-care providers in virtually all aspects of care for IPV/SV survivors essential for an adequate response.

Clinical Guidelines:

Women-centered care for IPV/SV survivors was especially strong in the important areas of privacy, confidentiality, danger assessment, safety planning, and supportive reactions to disclosure. Strength was also evident in providing written information and referring to legal, housing and economic empowerment services by a referral pathway. Women with special needs related to mental disorders and/or physical disability and referrals and referral pathways for services were also fairly well addressed. Moderate gaps were evident in selective enquiry for IPV, SV, assessing mental health (suicide, self-harm, depression, anxiety), providing mental health services, psychological first aid, watchful waiting to see if early distress resolves, providing or mobilizing social support, and structured advocacy and empowerment programs. Moderate gaps were also seen in assessing and providing for children’s safety, providing psychotherapy for children exposed to IPV and meeting the special needs of pregnant women, all of which are essential issues. Documentation with a body diagram or photo of injuries if present which may be essential for legal proceedings was also a moderate gap. Large gaps were evident in advising on coping strategies for stress and assessing for substance abuse and posttraumatic stress disorder. Large gaps were also present on advising women on the potential risks of taking home written materials, directories of existing services, shelters and hotlines, all of which are critical aspects of a healthcare response to IPV/SV. Unfortunately, the WHO guidance does not address IPV/SV in same sex relationships and as our project was to examine baseline adherence to the WHO Clinical and Policy Guidelines, we did not include this important topic in our scoring matrix.

There were additional strengths in the care of SV survivors; most guidelines appropriately did not require STI testing before treatment or HIV testing before post-exposure prophylaxis (PEP), advising about recording the time and type of assault, the need for vaginal swabs, the risks of pregnancy, STI’s, Hepatitis B and HIV and emergency contraception within five days of SV. Moderate gaps for survivors of SV were seen in the availability of sexual assault services available 24/7 and the need to collect anal and oral swabs and hair and fibers to assist in legal proceedings. Specific advice about types of emergency contraception, referral for abortion where legal, specific treatment for various STI’s, and offering hepatitis B vaccine were moderate gaps. HIV testing and counselling at first consultation, HIV-PEP appropriateness counselling, shared decision making, starting HIV PEP within 72 h, 2 or 3 drugs for HIV PEP, regular follow-ups, offering adherence counselling, and using drugs that followed national guidelines were also moderate gaps. Large gaps for SV survivors included prioritizing drugs with fewer side effects, although this decision may have been pre-empted by national drug guidelines.

The largest gaps noted were in the area of providing training to healthcare professionals who are often the most trusted and consulted by victims of violence. We asked about content, frequency and timing (in-service/prequalification) training, but assessing quality was outside the scope of this effort. Evidence suggests that “one-shot training” is not sufficient and trainings should be repeated/ongoing but this is not what occurs in low- and middle-income countries. The WHO Guidelines state “training should be intensive and content-appropriate to the context and setting. Intensive multidisciplinary training…should be offered to health care professionals where referrals to specialist domestic violence services are possible” (11: pp 35–36). Only half of the guidelines advised in-service training for frontline healthcare providers and provided information to them about IPV/SV and the best ways to respond to disclosure. Large gaps were reported on nearly all training items in the WHO Clinical Guidelines. None of the guidelines included training of prequalification healthcare workers or how direct healthcare providers should collect forensic evidence, so necessary for legal prosecution of perpetrators. Only one guideline suggested the length and frequency of training and there was poor psychological support for vicarious trauma among healthcare providers caring for IPV/SV patients. Clearly training is a crucial step in implementing the WHO Clinical and Policy Guidelines [11].

However, development of policies and guidelines is only the first step; monitoring and evaluating services for IPV/SV survivors are essential for implementation. Large gaps were reported in promotion of mechanisms and meetings for intersectoral collaboration and evaluation of services. To the best of our knowledge, there are no regional efforts to assess the implementation of health sector policies/protocols, though there may be some ongoing country-specific initiatives. The gap between policy, funding and implementation is key. PAHO is currently preparing a Regional Strategy and Plan of Action on strengthening health systems to address VAW to be reviewed and (hopefully) approved by Ministers of Health from 35 countries in the Americas. This document highlights the gap between policy and implementation and tries to push for advancements in terms of the creation of dedicated budget lines for VAW work within health budgets, for instance. Optimal evaluation should include intersectoral collaboration as well as input from women survivors of IPV/SV. Intersectoral collaboration is essential to improve policies and services, but also to change the public perception against IPV/SV especially in patriarchal societies where VAW may be tolerated or ignored.

## Conclusions

Baseline measurement of policy and clinical guidelines for IPV/SV in LAC PAHO/WHO member countries at the time of issuing the 2013 WHO Clinical and Policy Guidelines reveals some important strengths, but also serious gaps that need to be addressed. While there are specific topic gaps within women-centered care for IPV/SV survivors and additional issues for SV survivors, the most pressing needs are for concerted training initiatives for healthcare providers and strengthening multisectoral collaboration, monitoring and evaluation of services. A future evaluation of national policies, clinical guidelines, monitoring and evaluation will need to be conducted to measure the progress of the required scaling-up process.
